# Supplementary material for: Rapid turnover of life-cycle-related genes in the brown algae
Source: Genome Biol. 2019 Feb 14;20:35. doi: 10.1186/s13059-019-1630-6 (PMC6374913; doi:10.1186/s13059-019-1630-6)
Supplement: Supplementary file 3 — Protein alignments, corresponding Gblocks html file and CDS sequences for the lineage conserved gametophyte-biased genes. (ZIP 2109 kb) [file 13059_2019_1630_MOESM3_ESM.zip › Con_GA_alignments/Ec-22_001250.aa_ali.fasta-gb.htm]

Ec-22\_001250.aa.fasta.trans.aa\_ali.fasta


## Gblocks 0.91b Results

Processed file: **Ec-22\_001250.aa.fasta.trans.aa\_ali.fasta**  
Number of sequences: **2**  
Alignment assumed to be: **Protein**  
New number of positions: **383** (selected positions are underlined in blue)

```
                         10        20        30        40        50        60
                 =========+=========+=========+=========+=========+=========+
Ec-22_001250     MVPSLAGSSDLSSGVISGFRRALVWADGGQAAAAEGCPSVVSSLNGIPGRGAGSADVYSD
MSTRG.44146.1    ------------------------------------------------------------
                                                                             


                         70        80        90       100       110       120
                 =========+=========+=========+=========+=========+=========+
Ec-22_001250     NATGGEHADQSRNLPGEGNRDNHPKPAQGLHREGSGDNSDNDRDDDYALSSIGGGDDADG
MSTRG.44146.1    ---GGEP-----------------------------------------------------
                                                                             


                        130       140       150       160       170       180
                 =========+=========+=========+=========+=========+=========+
Ec-22_001250     RFGNAGGTHGRLSRGGGGGITSHDPEVKGVGAPEGDGYKLDGRLKESLTWRWPSMCSTAD
MSTRG.44146.1    ------------------------------------------------------------
                                                                             


                        190       200       210       220       230       240
                 =========+=========+=========+=========+=========+=========+
Ec-22_001250     ARGANDDLSPQKPPKGSYLRQRGPTDFRDFFDSEEEMAARQQQPKAYAHLGGLREEGKAK
MSTRG.44146.1    --------------SEQYYDASTRKDFRDFFDDEEEESARLQKPKAYAYLGGLREAGTAK
                                  ###########################################


                        250       260       270       280       290       300
                 =========+=========+=========+=========+=========+=========+
Ec-22_001250     YVFDRTSHT--RKGSLAAHLRGDYEPRPKGYRGTNVHSVADWRRLNTTLDRYAITGREAM
MSTRG.44146.1    YVFDRTSHSQAEKGALAAHLRGDYEPRASGYRGKNVHSVTDWRRPNTSLDKYAQSGRGAM
                 ########    ################################################


                        310       320       330       340       350       360
                 =========+=========+=========+=========+=========+=========+
Ec-22_001250     ERVAKSDRELRSQGVWVRQHTKAREQRRQRANHLLEVYRSELLKEQLREIEALHLAPGEQ
MSTRG.44146.1    ALVAESDRTLKSQGVWVRQQIRAREQRRQRANHMLEVYRSELRQEQLREIEVLRLPPREQ
                 ############################################################


                        370       380       390       400       410       420
                 =========+=========+=========+=========+=========+=========+
Ec-22_001250     HAHATKNMKDQILAADRIMRTVLRYGLVGNMEDAHYVKYAADAAQRAGLASTN-------
MSTRG.44146.1    RLRASKTMQDQITAADRIMRTIARYGLAGNMEDAHYIAYAADAAHRAGLTGDAEKKRTKI
                 #################################################           


                        430       440       450       460       470       480
                 =========+=========+=========+=========+=========+=========+
Ec-22_001250     ---GAAAAEMKAGGGSGAGGGTAR--ERPNGDGGVVKASSKKSARVATKKRAGSKHGVGD
MSTRG.44146.1    GRCGVGHPRRETASPEKLGGGTSGGPRRQRQQDGVPQADGKAPNGRKTD--RGVRHESGD
                                            #####################    ########


                        490       500       510       520       530       540
                 =========+=========+=========+=========+=========+=========+
Ec-22_001250     DSG-EQMSPYSVALARPLPARVTPDTERRTGQLKTAKPPLNAGRFALGEGQGTASAAAEQ
MSTRG.44146.1    DSADDEMSPYSLALTRPVPAAIQPKAKARGSTST-------TSR----TSRPTQNLAVTA
                 ##    #######################                       ########


                        550       560       570       580       590       600
                 =========+=========+=========+=========+=========+=========+
Ec-22_001250     ARRSLIQSERLRTFSLAGHPLRRGAENDTTAVPALIPIPEGFGHANGQHKSFVAGRIIEA
MSTRG.44146.1    ARRDNLQSERLRTFQLAGHPLRRGTEDDTTAVPALIPIPEGLGNANGEHKSYAAGRIVEA
                 ############################################################


                        610       620       630       640       650       660
                 =========+=========+=========+=========+=========+=========+
Ec-22_001250     LRASQVDAITGRQKRIAVPLWHRATDDMDSSGAGSVLYSDLIGHTYSVLVGPTRGVVGRR
MSTRG.44146.1    LRKSQVDSITGRRSKLAVPLWHRATEDLDS-GAGSVLYSDLIGHTYSVLVGPKR-----T
                 ############################## #######################      


                 
                 ====
Ec-22_001250     QGTG
MSTRG.44146.1    KETG
```

```
Parameters used
Minimum Number Of Sequences For A Conserved Position: 2
Minimum Number Of Sequences For A Flanking Position: 2
Maximum Number Of Contiguous Nonconserved Positions: 8
Minimum Length Of A Block: 10
Allowed Gap Positions: None
Use Similarity Matrices: Yes
```

```
Flank positions of the 7 selected block(s)
Flanks: [198  248]  [253  409]  [448  468]  [473  482]  [487  509]  [533  630]  [632  654]  

New number of positions in Ec-22_001250.aa.fasta.trans.aa_ali.fasta-gb:  383  (57% of the original 664 positions)
```
